# 2-Chloro-*N*-(3-chloro­benzo­yl)benzene­sulfonamide

**DOI:** 10.1107/S1600536810016909

**Published:** 2010-05-15

**Authors:** B. Thimme Gowda, Sabine Foro, P. A. Suchetan, Hartmut Fuess

**Affiliations:** a Department of Chemistry, Mangalore University, Mangalagangotri 574 199, Mangalore, India; b Institute of Materials Science, Darmstadt University of Technology, Petersenstrasse 23, D-64287 Darmstadt, Germany

## Abstract

The asymmetric unit of the title compound, C_13_H_9_Cl_2_NO_3_S, contains two independent mol­ecules. The conformation of the C=O bond is *anti* to the *meta*-Cl group in the chloro­benzoyl group of one of the mol­ecules and *syn* in the other. The dihedral angles between the sulfonyl and benzoyl benzene rings are 77.8 (1) and 83.5 (1)°. In the crystal structure, two pairs of independent mol­ecules are linked into a tetra­mer by N—H⋯O hydrogen bonds.

## Related literature

For background literature and related structures, see: Gowda *et al.* (2009 ▶, 2010 ▶); Suchetan *et al.* (2010**a* ▶,b* ▶).
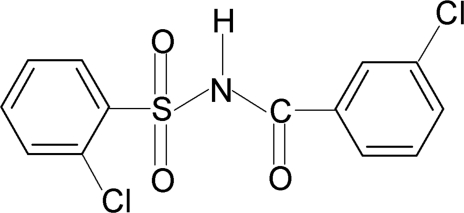


## Experimental

### 

#### Crystal data

C_13_H_9_Cl_2_NO_3_S
*M* *_r_* = 330.17
Triclinic, 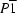

*a* = 7.4399 (6) Å
*b* = 11.679 (1) Å
*c* = 17.138 (2) Å
α = 75.346 (7)°
β = 83.188 (8)°
γ = 77.732 (7)°
*V* = 1404.5 (2) Å^3^
*Z* = 4
Mo *K*α radiation
μ = 0.62 mm^−1^
*T* = 299 K
0.20 × 0.14 × 0.08 mm

#### Data collection

Oxford Diffraction Xcalibur diffractometer with a Sapphire CCD detector
Absorption correction: multi-scan (*CrysAlis RED*; Oxford Diffraction, 2009 ▶) *T*_min_ = 0.887, *T*_max_ = 0.952
9768 measured reflections
5694 independent reflections
4267 reflections with *I* > 2σ(*I*)
*R*_int_ = 0.018

#### Refinement

*R*[*F* ^2^ > 2σ(*F* ^2^)] = 0.054
*wR*(*F* ^2^) = 0.154
*S* = 1.04
5694 reflections
367 parameters
2 restraints
H atoms treated by a mixture of independent and constrained refinement
Δρ_max_ = 1.10 e Å^−3^
Δρ_min_ = −0.50 e Å^−3^

### 

Data collection: *CrysAlis CCD* (Oxford Diffraction, 2009 ▶); cell refinement: *CrysAlis RED* (Oxford Diffraction, 2009 ▶); data reduction: *CrysAlis RED*; program(s) used to solve structure: *SHELXS97* (Sheldrick, 2008 ▶); program(s) used to refine structure: *SHELXL97* (Sheldrick, 2008 ▶); molecular graphics: *PLATON* (Spek, 2009 ▶); software used to prepare material for publication: *SHELXL97*.

## Supplementary Material

Crystal structure: contains datablocks I, global. DOI: 10.1107/S1600536810016909/ci5091sup1.cif

Structure factors: contains datablocks I. DOI: 10.1107/S1600536810016909/ci5091Isup2.hkl

Additional supplementary materials:  crystallographic information; 3D view; checkCIF report

## Figures and Tables

**Table 1:** Hydrogen-bond geometry (Å, °)

*D*—H⋯*A*	*D*—H	H⋯*A*	*D*⋯*A*	*D*—H⋯*A*
N1—H1*N*⋯O6	0.84 (2)	2.02 (2)	2.836 (4)	162 (4)
N2—H2*N*⋯O5^i^	0.85 (2)	2.10 (2)	2.937 (4)	173 (4)

Symmetry code: (i) 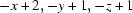.

**Fig. 1.**
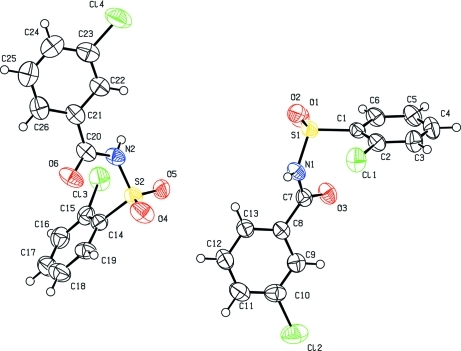
The two independent molecules of the title compound, showing the atom-labelling scheme. Displacement ellipsoids are drawn at the 50% probability level.

## supplementary crystallographic information

### Comment

As a part of studying the effect of ring and the side chain substitutions on the crystal structures of *N*-aryl sulfonamides (Gowda *et al.*, 2009, 2010; Suchetan *et al.*, 2010*a,b*), the structure of 2-chloro-*N*-(3-chlorobenzoyl)-benzenesulfonamide (I) has been determined. The asymmetric unit of (I) contains two independent molecules. In the C—SO_2_—NH—C(O) segments, the N—H bonds are *anti* to the C═O bonds (Fig.1), similar to those observed in 2-chloro-*N*- (3-methylbenzoyl)-benzenesulfonamide (II) (Suchetan *et al.*, 2010*b*), 2-methyl-*N*-(3-methylbenzoyl)-benzenesulfonamide (III) (Gowda *et al.*, 2010), 2-chloro-*N*-(2-chlorobenzoyl)- benzenesulfonamide (IV)(Suchetan *et al.*, 2010*a*), *N*-(benzoyl)-benzenesulfonamide (V) (Gowda *et al.*, 2009).

The conformation of the C═O bond is *anti* to the *meta*-Cl group in the benzoyl ring of one of the molecules and *syn* in the other, compared to the *anti* conformation observed between C═O bond and *meta*- methyl group in the benzoyl ring of (II).

The chlorobenzoyl and sulfonyl-bound chlorophenyl units in the two molecules of (I) are twisted with respect to the S—N bond, with torsional angles of -62.6 (3)° [C7—N1—S1—C1] and -62.6 (3)° [C20—N2—S2—C14], compared to  those of -66.5 (2)° in (II), -66.2 (3)° in (III), -66.5 (2)° in (IV) and -66.9 (3)° in (V). The dihedral angles between the sulfonyl and the benzoyl- bound benzene rings are 77.8 (1)° (molecule 1) and 83.5 (1)° (molecule 2), compared to the values of 74.7 (1)° in (II), 74.8 (1)° in (III), 76.9 (1) in (IV) and 80.3 (1)° in (V).

### Experimental

The title compound was prepared by refluxing a mixture of 3-chlorobenzoic acid, 2-chlorobenzenesulfonamide and phosphorous oxy chloride for 3 h on a water bath. The resultant mixture was cooled and poured into ice cold water. The solid obtained was filtered, washed thoroughly with water and then dissolved in sodium bicarbonate solution. The compound was later reprecipitated by acidifying the filtered solution with dilute HCl. It was filtered, dried and recrystallized. Rod like colourless single crystals of the title compound used in X-ray diffraction studies were obtained by a slow evaporation of its toluene solution at room temperature.

### Refinement

The H atoms of the NH groups were located in a difference map and refined with the a N–H distance restraint of 0.86 (2) %A. The other H atoms were positioned with idealized geometry using a riding model with C—H = 0.93 Å.

All H atoms were refined with isotropic displacement parameters (set to 1.2 times of the *U*_eq_ of the parent atom). The residual electron-density features are located in the region of H25 and Cl4. The highest peak is 0.98 Å from H25 and the deepest hole is 0.68 Å from Cl4.

### Crystal data

**Table:**

C_13_H_9_Cl_2_NO_3_S	*Z* = 4
*M**_r_* = 330.17	*F*(000) = 672
Triclinic, *P*1	*D*_x_ = 1.561 Mg m^−^^3^
Hall symbol: -P 1	Mo *K*α radiation, λ = 0.71073 Å
*a* = 7.4399 (6) Å	Cell parameters from 3572 reflections
*b* = 11.679 (1) Å	θ = 2.5–27.8°
*c* = 17.138 (2) Å	µ = 0.62 mm^−^^1^
α = 75.346 (7)°	*T* = 299 K
β = 83.188 (8)°	Rod, colourless
γ = 77.732 (7)°	0.20 × 0.14 × 0.08 mm
*V* = 1404.5 (2) Å^3^	

### Data collection

**Table:**

Oxford Diffraction Xcalibur diffractometer with a Sapphire CCD detector	5694 independent reflections
Radiation source: fine-focus sealed tube	4267 reflections with *I* > 2σ(*I*)
graphite	*R*_int_ = 0.018
Rotation method data acquisition using ω and φ scans	θ_max_ = 26.4°, θ_min_ = 2.5°
Absorption correction: multi-scan (*CrysAlis RED*; Oxford Diffraction, 2009)	*h* = −9→5
*T*_min_ = 0.887, *T*_max_ = 0.952	*k* = −14→14
9768 measured reflections	*l* = −19→21

### Refinement

**Table:**

Refinement on *F*^2^	Primary atom site location: structure-invariant direct methods
Least-squares matrix: full	Secondary atom site location: difference Fourier map
*R*[*F*^2^ > 2σ(*F*^2^)] = 0.054	Hydrogen site location: inferred from neighbouring sites
*wR*(*F*^2^) = 0.154	H atoms treated by a mixture of independent and constrained refinement
*S* = 1.04	*w* = 1/[σ^2^(*F*_o_^2^) + (0.0682*P*)^2^ + 1.64*P*] where *P* = (*F*_o_^2^ + 2*F*_c_^2^)/3
5694 reflections	(Δ/σ)_max_ = 0.014
367 parameters	Δρ_max_ = 1.10 e Å^−^^3^
2 restraints	Δρ_min_ = −0.50 e Å^−^^3^

### Special details

**Table:**

Geometry. All esds (except the esd in the dihedral angle between two l.s. planes) are estimated using the full covariance matrix. The cell esds are taken into account individually in the estimation of esds in distances, angles and torsion angles; correlations between esds in cell parameters are only used when they are defined by crystal symmetry. An approximate (isotropic) treatment of cell esds is used for estimating esds involving l.s. planes.
Refinement. Refinement of *F*^2^ against ALL reflections. The weighted *R*-factor wR and goodness of fit *S* are based on *F*^2^, conventional *R*-factors *R* are based on *F*, with *F* set to zero for negative *F*^2^. The threshold expression of *F*^2^ > σ(*F*^2^) is used only for calculating *R*-factors(gt) etc. and is not relevant to the choice of reflections for refinement. *R*-factors based on *F*^2^ are statistically about twice as large as those based on *F*, and *R*- factors based on ALL data will be even larger.

### Fractional atomic coordinates and isotropic or equivalent isotropic displacement parameters (Å^2^)

**Table:**

	*x*	*y*	*z*	*U*_iso_*/*U*_eq_	
C1	0.2455 (4)	1.0034 (3)	0.13610 (19)	0.0380 (7)	
C2	0.4149 (4)	1.0391 (3)	0.1159 (2)	0.0432 (8)	
C3	0.4422 (5)	1.1225 (3)	0.0442 (2)	0.0543 (9)	
H3	0.5556	1.1464	0.0304	0.065*	
C4	0.3009 (6)	1.1700 (4)	−0.0067 (2)	0.0594 (10)	
H4	0.3199	1.2258	−0.0548	0.071*	
C5	0.1320 (5)	1.1358 (3)	0.0128 (2)	0.0564 (10)	
H5	0.0372	1.1690	−0.0217	0.068*	
C6	0.1041 (5)	1.0522 (3)	0.0839 (2)	0.0473 (8)	
H6	−0.0095	1.0283	0.0970	0.057*	
C7	0.3347 (4)	0.7053 (3)	0.1700 (2)	0.0421 (7)	
C8	0.4322 (4)	0.5771 (3)	0.1922 (2)	0.0403 (7)	
C9	0.5166 (5)	0.5225 (3)	0.1304 (2)	0.0488 (8)	
H9	0.5157	0.5659	0.0768	0.059*	
C10	0.6012 (5)	0.4034 (4)	0.1500 (3)	0.0539 (9)	
C11	0.5995 (5)	0.3361 (4)	0.2282 (3)	0.0596 (10)	
H11	0.6549	0.2550	0.2399	0.072*	
C12	0.5148 (6)	0.3901 (4)	0.2892 (3)	0.0614 (10)	
H12	0.5135	0.3450	0.3424	0.074*	
C13	0.4315 (5)	0.5106 (3)	0.2723 (2)	0.0487 (8)	
H13	0.3757	0.5468	0.3138	0.058*	
N1	0.3355 (4)	0.7713 (2)	0.22646 (18)	0.0434 (7)	
H1N	0.423 (4)	0.755 (3)	0.2573 (19)	0.052*	
O1	0.0127 (3)	0.8835 (2)	0.22699 (17)	0.0600 (7)	
O2	0.2495 (4)	0.9355 (2)	0.29433 (14)	0.0529 (6)	
O3	0.2575 (4)	0.7488 (2)	0.10731 (15)	0.0548 (6)	
S1	0.19546 (11)	0.90062 (8)	0.22791 (5)	0.0418 (2)	
Cl1	0.59757 (13)	0.98058 (10)	0.17710 (6)	0.0621 (3)	
Cl2	0.71150 (19)	0.33696 (12)	0.07272 (9)	0.0864 (4)	
C14	0.7395 (4)	0.7963 (3)	0.4444 (2)	0.0418 (7)	
C15	0.9034 (5)	0.8353 (3)	0.4444 (2)	0.0469 (8)	
C16	0.9072 (6)	0.9573 (4)	0.4233 (2)	0.0573 (10)	
H16	1.0162	0.9838	0.4240	0.069*	
C17	0.7485 (7)	1.0391 (4)	0.4014 (3)	0.0648 (11)	
H17	0.7510	1.1211	0.3875	0.078*	
C18	0.5875 (6)	1.0018 (4)	0.3996 (3)	0.0633 (11)	
H18	0.4816	1.0582	0.3841	0.076*	
C19	0.5817 (5)	0.8804 (3)	0.4208 (2)	0.0506 (9)	
H19	0.4722	0.8550	0.4193	0.061*	
C20	0.7982 (5)	0.6143 (3)	0.3232 (2)	0.0473 (8)	
C21	0.9079 (5)	0.5420 (3)	0.2688 (2)	0.0470 (8)	
C22	0.9803 (5)	0.4209 (3)	0.2962 (2)	0.0511 (9)	
H22	0.9681	0.3831	0.3507	0.061*	
C23	1.0719 (5)	0.3567 (4)	0.2401 (3)	0.0597 (10)	
C24	1.0906 (6)	0.4131 (5)	0.1593 (3)	0.0659 (11)	
H24	1.1511	0.3691	0.1225	0.079*	
C25	1.0212 (6)	0.5326 (5)	0.1332 (3)	0.0721 (12)	
H25	1.0363	0.5703	0.0788	0.087*	
C26	0.9289 (5)	0.5978 (4)	0.1869 (2)	0.0587 (10)	
H26	0.8803	0.6793	0.1687	0.070*	
N2	0.8416 (4)	0.5803 (3)	0.40323 (18)	0.0477 (7)	
H2N	0.946 (3)	0.537 (3)	0.415 (2)	0.057*	
O4	0.5315 (3)	0.6379 (3)	0.4726 (2)	0.0700 (8)	
O5	0.8139 (3)	0.5845 (2)	0.54567 (15)	0.0547 (6)	
O6	0.6750 (4)	0.6993 (2)	0.30102 (18)	0.0620 (8)	
S2	0.71956 (11)	0.64315 (8)	0.47362 (6)	0.0466 (2)	
Cl3	1.10996 (13)	0.73583 (10)	0.46678 (7)	0.0686 (3)	
Cl4	1.1539 (2)	0.20474 (11)	0.27335 (10)	0.0915 (4)	

### Atomic displacement parameters (Å^2^)

**Table:**

	*U*^11^	*U*^22^	*U*^33^	*U*^12^	*U*^13^	*U*^23^
C1	0.0388 (16)	0.0328 (16)	0.0392 (17)	−0.0003 (13)	−0.0070 (13)	−0.0059 (13)
C2	0.0401 (18)	0.0421 (18)	0.0462 (19)	−0.0046 (14)	−0.0122 (14)	−0.0067 (15)
C3	0.054 (2)	0.054 (2)	0.053 (2)	−0.0187 (18)	−0.0060 (17)	−0.0012 (17)
C4	0.075 (3)	0.048 (2)	0.049 (2)	−0.0125 (19)	−0.0116 (19)	0.0042 (17)
C5	0.058 (2)	0.050 (2)	0.053 (2)	−0.0010 (18)	−0.0241 (18)	0.0023 (17)
C6	0.0389 (18)	0.0459 (19)	0.055 (2)	−0.0031 (15)	−0.0151 (15)	−0.0060 (16)
C7	0.0380 (17)	0.0400 (18)	0.0467 (19)	−0.0116 (14)	−0.0031 (14)	−0.0040 (14)
C8	0.0340 (16)	0.0381 (17)	0.0496 (19)	−0.0107 (13)	−0.0064 (14)	−0.0067 (14)
C9	0.050 (2)	0.048 (2)	0.051 (2)	−0.0133 (16)	−0.0048 (16)	−0.0118 (16)
C10	0.047 (2)	0.051 (2)	0.069 (3)	−0.0105 (17)	−0.0015 (18)	−0.0247 (19)
C11	0.054 (2)	0.042 (2)	0.078 (3)	−0.0032 (17)	−0.006 (2)	−0.0086 (19)
C12	0.063 (2)	0.047 (2)	0.062 (3)	−0.0045 (19)	−0.003 (2)	0.0032 (19)
C13	0.0467 (19)	0.0434 (19)	0.052 (2)	−0.0079 (15)	−0.0009 (16)	−0.0050 (16)
N1	0.0409 (15)	0.0375 (15)	0.0500 (17)	−0.0019 (12)	−0.0174 (12)	−0.0052 (13)
O1	0.0351 (13)	0.0638 (17)	0.0706 (18)	−0.0073 (12)	−0.0041 (12)	0.0016 (13)
O2	0.0630 (16)	0.0529 (15)	0.0388 (13)	−0.0027 (12)	−0.0037 (11)	−0.0104 (11)
O3	0.0632 (16)	0.0486 (14)	0.0504 (15)	−0.0082 (12)	−0.0212 (12)	−0.0019 (11)
S1	0.0359 (4)	0.0405 (4)	0.0432 (5)	−0.0018 (3)	−0.0037 (3)	−0.0033 (3)
Cl1	0.0421 (5)	0.0781 (7)	0.0618 (6)	−0.0153 (4)	−0.0194 (4)	0.0017 (5)
Cl2	0.0947 (9)	0.0792 (8)	0.0946 (9)	−0.0088 (7)	0.0062 (7)	−0.0494 (7)
C14	0.0397 (17)	0.0442 (18)	0.0381 (17)	−0.0008 (14)	−0.0055 (13)	−0.0082 (14)
C15	0.0420 (18)	0.058 (2)	0.0384 (18)	−0.0080 (16)	−0.0051 (14)	−0.0075 (16)
C16	0.063 (2)	0.062 (2)	0.051 (2)	−0.022 (2)	−0.0014 (18)	−0.0133 (18)
C17	0.087 (3)	0.046 (2)	0.059 (2)	−0.008 (2)	−0.006 (2)	−0.0116 (18)
C18	0.066 (3)	0.052 (2)	0.067 (3)	0.008 (2)	−0.017 (2)	−0.014 (2)
C19	0.0440 (19)	0.052 (2)	0.056 (2)	0.0047 (16)	−0.0138 (16)	−0.0199 (17)
C20	0.0471 (19)	0.0395 (18)	0.059 (2)	−0.0079 (15)	−0.0202 (16)	−0.0107 (16)
C21	0.0423 (18)	0.0457 (19)	0.056 (2)	−0.0091 (15)	−0.0205 (16)	−0.0082 (16)
C22	0.052 (2)	0.046 (2)	0.058 (2)	−0.0081 (16)	−0.0127 (17)	−0.0133 (17)
C23	0.051 (2)	0.051 (2)	0.083 (3)	−0.0113 (18)	−0.012 (2)	−0.022 (2)
C24	0.057 (2)	0.086 (3)	0.066 (3)	−0.024 (2)	0.001 (2)	−0.032 (2)
C25	0.072 (3)	0.086 (3)	0.060 (3)	−0.027 (3)	−0.006 (2)	−0.010 (2)
C26	0.056 (2)	0.060 (2)	0.060 (2)	−0.0166 (19)	−0.0180 (19)	−0.0027 (19)
N2	0.0421 (16)	0.0456 (17)	0.0520 (18)	0.0066 (13)	−0.0206 (13)	−0.0100 (13)
O4	0.0343 (13)	0.0585 (17)	0.112 (2)	−0.0072 (12)	−0.0075 (14)	−0.0100 (16)
O5	0.0500 (14)	0.0572 (15)	0.0441 (14)	0.0009 (12)	−0.0030 (11)	0.0020 (11)
O6	0.0612 (16)	0.0461 (15)	0.0787 (19)	0.0104 (12)	−0.0416 (14)	−0.0149 (13)
S2	0.0334 (4)	0.0445 (5)	0.0555 (5)	−0.0009 (3)	−0.0062 (4)	−0.0041 (4)
Cl3	0.0357 (5)	0.0757 (7)	0.0822 (7)	−0.0091 (4)	−0.0112 (4)	0.0057 (5)
Cl4	0.1048 (10)	0.0482 (6)	0.1177 (11)	0.0030 (6)	−0.0070 (8)	−0.0277 (7)

### Geometric parameters (Å, °)

**Table:**

C1—C2	1.389 (5)	C14—C19	1.388 (5)
C1—C6	1.394 (4)	C14—C15	1.390 (5)
C1—S1	1.775 (3)	C14—S2	1.765 (4)
C2—C3	1.386 (5)	C15—C16	1.384 (5)
C2—Cl1	1.730 (3)	C15—Cl3	1.736 (4)
C3—C4	1.377 (5)	C16—C17	1.375 (6)
C3—H3	0.93	C16—H16	0.93
C4—C5	1.377 (6)	C17—C18	1.365 (6)
C4—H4	0.93	C17—H17	0.93
C5—C6	1.381 (5)	C18—C19	1.380 (6)
C5—H5	0.93	C18—H18	0.93
C6—H6	0.93	C19—H19	0.93
C7—O3	1.220 (4)	C20—O6	1.214 (4)
C7—N1	1.382 (4)	C20—N2	1.384 (5)
C7—C8	1.493 (5)	C20—C21	1.475 (5)
C8—C9	1.391 (5)	C21—C22	1.382 (5)
C8—C13	1.396 (5)	C21—C26	1.396 (5)
C9—C10	1.374 (5)	C22—C23	1.393 (6)
C9—H9	0.93	C22—H22	0.93
C10—C11	1.372 (6)	C23—C24	1.379 (6)
C10—Cl2	1.738 (4)	C23—Cl4	1.719 (4)
C11—C12	1.377 (6)	C24—C25	1.359 (7)
C11—H11	0.93	C24—H24	0.93
C12—C13	1.383 (5)	C25—C26	1.371 (6)
C12—H12	0.93	C25—H25	0.93
C13—H13	0.93	C26—H26	0.93
N1—S1	1.647 (3)	N2—S2	1.650 (3)
N1—H1N	0.845 (18)	N2—H2N	0.846 (19)
O1—S1	1.418 (3)	O4—S2	1.416 (3)
O2—S1	1.427 (3)	O5—S2	1.435 (3)
			
C2—C1—C6	119.5 (3)	C19—C14—C15	119.4 (3)
C2—C1—S1	123.1 (2)	C19—C14—S2	117.3 (3)
C6—C1—S1	117.3 (3)	C15—C14—S2	123.2 (3)
C3—C2—C1	119.9 (3)	C16—C15—C14	120.0 (3)
C3—C2—Cl1	118.1 (3)	C16—C15—Cl3	117.6 (3)
C1—C2—Cl1	122.0 (3)	C14—C15—Cl3	122.4 (3)
C4—C3—C2	119.9 (4)	C17—C16—C15	119.5 (4)
C4—C3—H3	120.1	C17—C16—H16	120.2
C2—C3—H3	120.1	C15—C16—H16	120.2
C5—C4—C3	120.8 (4)	C18—C17—C16	121.0 (4)
C5—C4—H4	119.6	C18—C17—H17	119.5
C3—C4—H4	119.6	C16—C17—H17	119.5
C4—C5—C6	119.7 (3)	C17—C18—C19	120.0 (4)
C4—C5—H5	120.1	C17—C18—H18	120.0
C6—C5—H5	120.1	C19—C18—H18	120.0
C5—C6—C1	120.2 (3)	C18—C19—C14	120.0 (4)
C5—C6—H6	119.9	C18—C19—H19	120.0
C1—C6—H6	119.9	C14—C19—H19	120.0
O3—C7—N1	122.1 (3)	O6—C20—N2	120.3 (3)
O3—C7—C8	122.8 (3)	O6—C20—C21	123.3 (3)
N1—C7—C8	115.0 (3)	N2—C20—C21	116.4 (3)
C9—C8—C13	120.1 (3)	C22—C21—C26	120.2 (4)
C9—C8—C7	118.4 (3)	C22—C21—C20	121.8 (3)
C13—C8—C7	121.4 (3)	C26—C21—C20	117.9 (3)
C10—C9—C8	118.8 (4)	C21—C22—C23	118.5 (4)
C10—C9—H9	120.6	C21—C22—H22	120.8
C8—C9—H9	120.6	C23—C22—H22	120.8
C9—C10—C11	121.9 (4)	C24—C23—C22	120.6 (4)
C9—C10—Cl2	118.5 (3)	C24—C23—Cl4	120.8 (4)
C11—C10—Cl2	119.6 (3)	C22—C23—Cl4	118.6 (3)
C12—C11—C10	119.2 (4)	C25—C24—C23	120.5 (4)
C12—C11—H11	120.4	C25—C24—H24	119.7
C10—C11—H11	120.4	C23—C24—H24	119.7
C11—C12—C13	120.8 (4)	C24—C25—C26	120.2 (4)
C11—C12—H12	119.6	C24—C25—H25	119.9
C13—C12—H12	119.6	C26—C25—H25	119.9
C12—C13—C8	119.2 (4)	C25—C26—C21	120.1 (4)
C12—C13—H13	120.4	C25—C26—H26	119.9
C8—C13—H13	120.4	C21—C26—H26	119.9
C7—N1—S1	123.8 (2)	C20—N2—S2	122.5 (2)
C7—N1—H1N	121 (3)	C20—N2—H2N	119 (3)
S1—N1—H1N	114 (3)	S2—N2—H2N	117 (3)
O1—S1—O2	120.43 (17)	O4—S2—O5	119.14 (18)
O1—S1—N1	107.77 (16)	O4—S2—N2	109.45 (18)
O2—S1—N1	103.83 (15)	O5—S2—N2	104.14 (15)
O1—S1—C1	107.48 (16)	O4—S2—C14	107.70 (16)
O2—S1—C1	109.82 (15)	O5—S2—C14	110.08 (16)
N1—S1—C1	106.73 (15)	N2—S2—C14	105.52 (16)
			
C6—C1—C2—C3	−0.2 (5)	C19—C14—C15—C16	2.0 (5)
S1—C1—C2—C3	−178.1 (3)	S2—C14—C15—C16	−177.8 (3)
C6—C1—C2—Cl1	−179.0 (3)	C19—C14—C15—Cl3	−175.8 (3)
S1—C1—C2—Cl1	3.1 (4)	S2—C14—C15—Cl3	4.5 (4)
C1—C2—C3—C4	0.2 (6)	C14—C15—C16—C17	−1.0 (6)
Cl1—C2—C3—C4	179.1 (3)	Cl3—C15—C16—C17	176.9 (3)
C2—C3—C4—C5	0.2 (6)	C15—C16—C17—C18	−0.4 (6)
C3—C4—C5—C6	−0.6 (6)	C16—C17—C18—C19	0.6 (7)
C4—C5—C6—C1	0.7 (6)	C17—C18—C19—C14	0.4 (6)
C2—C1—C6—C5	−0.3 (5)	C15—C14—C19—C18	−1.7 (5)
S1—C1—C6—C5	177.7 (3)	S2—C14—C19—C18	178.1 (3)
O3—C7—C8—C9	31.9 (5)	O6—C20—C21—C22	−149.0 (4)
N1—C7—C8—C9	−149.0 (3)	N2—C20—C21—C22	30.7 (5)
O3—C7—C8—C13	−144.7 (4)	O6—C20—C21—C26	27.5 (5)
N1—C7—C8—C13	34.4 (4)	N2—C20—C21—C26	−152.8 (3)
C13—C8—C9—C10	−1.1 (5)	C26—C21—C22—C23	−0.7 (5)
C7—C8—C9—C10	−177.8 (3)	C20—C21—C22—C23	175.8 (3)
C8—C9—C10—C11	2.2 (6)	C21—C22—C23—C24	0.4 (6)
C8—C9—C10—Cl2	−178.3 (3)	C21—C22—C23—Cl4	−177.4 (3)
C9—C10—C11—C12	−1.7 (6)	C22—C23—C24—C25	0.5 (6)
Cl2—C10—C11—C12	178.8 (3)	Cl4—C23—C24—C25	178.3 (3)
C10—C11—C12—C13	0.2 (6)	C23—C24—C25—C26	−1.1 (7)
C11—C12—C13—C8	0.8 (6)	C24—C25—C26—C21	0.8 (6)
C9—C8—C13—C12	−0.3 (5)	C22—C21—C26—C25	0.1 (5)
C7—C8—C13—C12	176.3 (3)	C20—C21—C26—C25	−176.5 (3)
O3—C7—N1—S1	15.6 (5)	O6—C20—N2—S2	5.7 (5)
C8—C7—N1—S1	−163.5 (2)	C21—C20—N2—S2	−173.9 (2)
C7—N1—S1—O1	52.6 (3)	C20—N2—S2—O4	53.1 (3)
C7—N1—S1—O2	−178.6 (3)	C20—N2—S2—O5	−178.5 (3)
C7—N1—S1—C1	−62.6 (3)	C20—N2—S2—C14	−62.6 (3)
C2—C1—S1—O1	−178.8 (3)	C19—C14—S2—O4	−4.5 (3)
C6—C1—S1—O1	3.3 (3)	C15—C14—S2—O4	175.2 (3)
C2—C1—S1—O2	48.6 (3)	C19—C14—S2—O5	−135.9 (3)
C6—C1—S1—O2	−129.4 (3)	C15—C14—S2—O5	43.9 (3)
C2—C1—S1—N1	−63.4 (3)	C19—C14—S2—N2	112.3 (3)
C6—C1—S1—N1	118.7 (3)	C15—C14—S2—N2	−67.9 (3)

### Hydrogen-bond geometry (Å, °)

**Table:**

*D*—H···*A*	*D*—H	H···*A*	*D*···*A*	*D*—H···*A*
N1—H1N···O6	0.84 (2)	2.02 (2)	2.836 (4)	162 (4)
N2—H2N···O5^i^	0.85 (2)	2.10 (2)	2.937 (4)	173 (4)

Symmetry codes: (i) −*x*+2, −*y*+1, −*z*+1.
